# Validated RP-HPLC method for simultaneous determination and quantification of chlorpheniramine maleate, paracetamol and caffeine in tablet formulation

**DOI:** 10.1186/s40064-016-2241-2

**Published:** 2016-05-14

**Authors:** Akwasi Acheampong, Wilfred Owusu Gyasi, Godfred Darko, Joseph Apau, Sylvester Addai-Arhin

**Affiliations:** Department of Chemistry, Kwame Nkrumah University of Science and Technology, Kumasi, Ghana; Department of Pharmaceutical Science, Faculty of Medicine and Health Science, Kumasi Polytechnic, Kumasi, Ghana

**Keywords:** Chlorpheniramine maleate, Paracetamol, Caffeine, RP-HPLC, Tablet formulations, Cyanocobalamin, Multi-component formulation

## Abstract

Chlorpheniramine maleate–paracetamol–caffeine tablet formulation is one of the common over-the-counter drugs used for the treatment of cold and cough. A reversed-phase high-performance liquid-chromatography method has been successfully developed for the simultaneous determination of chlorpheniramine maleate, paracetamol and caffeine in a drug formulation. The RP-HPLC method employed a Phenomenex C18 reversed phase column (Luna 5µ, 250 × 4.6 mm) with an isocratic mixture of methanol and 0.05 M dibasic phosphate buffer pH 4.0 in the ratio of (30:70; v/v) as the mobile phase. The column temperature was kept at 30 °C. The flow rate was 1.0 mL/min and detection was by means of a UV detector at wavelength of 215 nm. All the active components were successfully eluted with mean retention times of 2.4, 4.2, 7.2 min for chlorpheniramine maleate, paracetamol and caffeine respectively. The method was found to be linear (R^2^ > 0.99), precise (RSD < 2.0 %), accurate (recoveries 97.9–102.8 %), specific, simple, sensitive, rapid and robust. The validated method can be used in routine quality control analysis of fixed dose combination tablets containing chlorpheniramine maleate, paracetamol and caffeine without any interference by excipients.

## Background

Drug combinations are single preparations containing two or more active pharmaceutical ingredients (APIs) for the purpose of their concurrent administration as a fixed dose mixture combinations drug. Most multi-component drug formulations usually contain two or more active ingredients which are responsible for a combined therapeutic activity of the drug. This concept is beneficial when the selective agents have different mechanisms of action that provide additive or synergistic efficacy (Li et al. 2010). There is increased production of multicomponent drugs formulation due to increased efficacy, increased resistance of microorganisms to single component formulations and dependency and/or tolerance, and this has further led to increased drug counterfeiting and adulteration (Mackey and Liang 2011; Newton et al. 2006).

However, monographs in most official pharmacopoeia are for single component drugs, hence local Pharmaceutical manufacturing companies in the analysis of multi-component drug formulations use methods that involve multiple and repeated extractions to extract each active component before their quantification using spectrophotometry or titrimetry. Such methods are thus laborious and cumbersome. This has led to researchers developing various methods to help facilitate easy and quick analysis of multi-component drugs. With HPLC being a method of choice, many researchers have worked at developing various RP-HPLC methods for the simultaneous estimation of various active components in multi-component drugs (Sawyer and Kumar 2003; Okine et al. 2006; Cesar et al. 2008; Suresh et al. 2010; Tsvetkova et al. 2012; Chandra and Sharma 2013; Malakar et al. 2013, Acheampong et al. 2015).

There are many multi-component tablet formulation available in the market that contain chlorpheniramine maleate (CPM), paracetamol (PARA) and caffeine (CAF) as the only active components or as part of the many active components of the drug. Such drugs are usually used against nausea and motion sickness, common cold, and cough (Sawant and Borkar 2012). For this reason, researchers have worked at developing methods that can be used to simultaneously identify and quantify some or all the active ingredients in tablet formulations. Various analytical techniques have been employed in such methods including voltammetry (Shekappa et al. 2011; Saeed and Reyhaneh-Sadat 2011), fluorimetry (Hossein and Yahya 2011), colorimetry (Shihana et al. 2010), UV-spectrophotometry (Hadad et al. 2007; Ghulam et al. 2011; Khoshayand et al. 2010; Maryam and Mehdi 2005; Kuldeep et al. 2011), quantitative thin-layer chromatography (TLC) (Atul et al. 2008; Misra et al. 2009), high-performance liquid chromatography (HPLC) (Heydari 2008; Prasanna and Reddy 2009; Godse et al. 2009; Franeta et al. 2002; Pattan et al. 2009; Suryan et al. 2011; Viswanath et al. 2011), gas chromatography (GC) (Belal et al. 2009), RP-HPLC (Mukesh et al. 2010; Şenyuva and Özden 2002), and LC–MS–MS (Lou et al. 2010).

In developing countries where the challenge of sub-standard drugs and drug counterfeiting is enormous, there is the need for methods which are accurate, cost effective, easy to use, rapid and require the use of non-sophisticated equipment in order to facilitate easy identification and quantitation of the active components in multi-component formulations. The main objective of this work, therefore, is to develop and validate a new, simple, accurate, linear, precise, specific, robust, sensitive and cost effective RP-HPLC method for simultaneous estimation of chlorpheniramine maleate, paracetamol and caffeine in multi-component tablets.

## Results and discussion

### Method development and optimization

#### Mobile phase selection

**Fig. 1:**
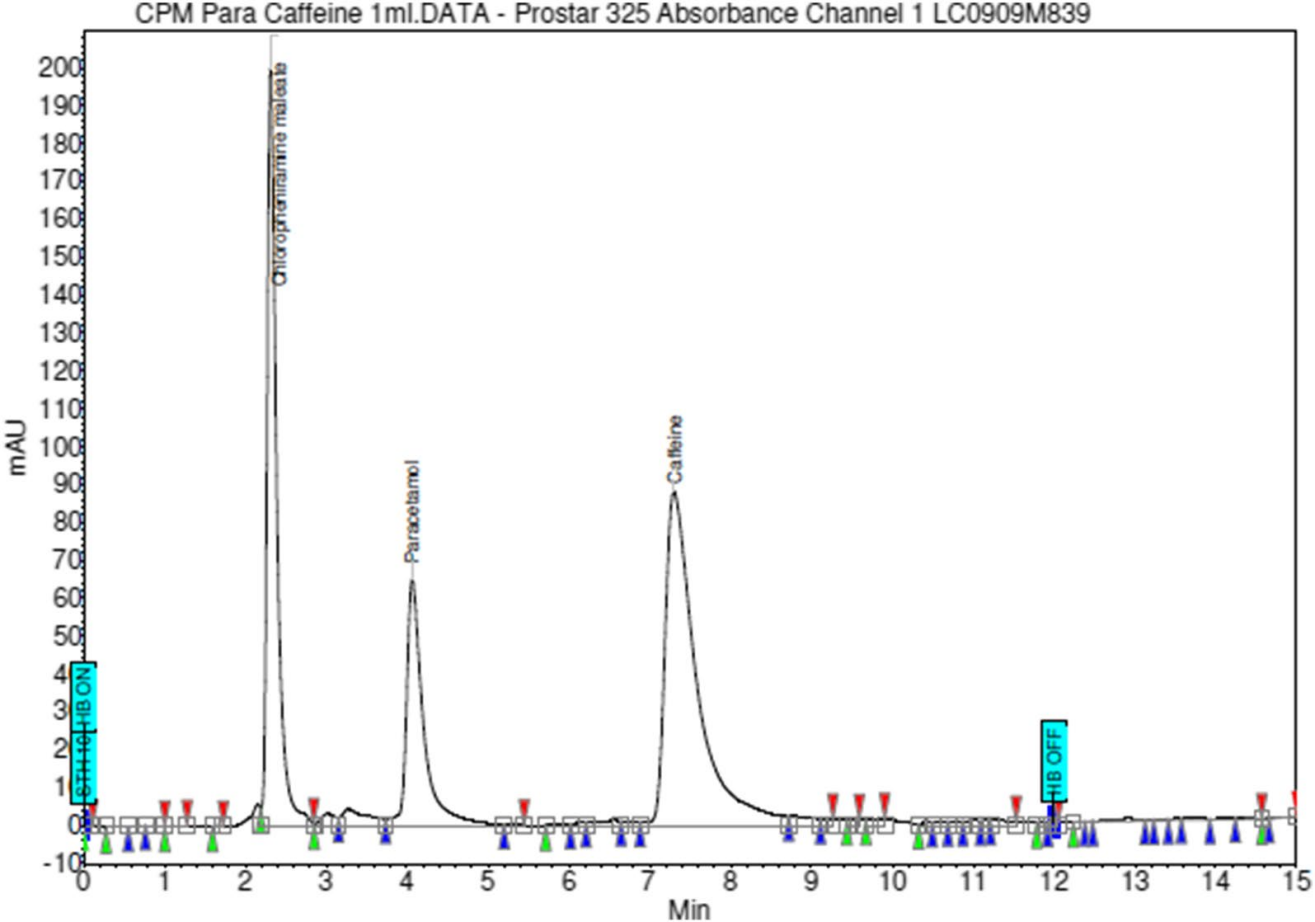
Optimized chromatogram of single injection of solution containing standard of chlorpheniramine maleate, paracetamol, and caffeine

Preliminary studies with several solvent systems were performed to select the most effective solvent system for the separation of the three APIs. The selection of these solvents as possible mobile phase(s) depended on factors such as cost of solvent(s), polarities of solvent(s) and that of the analyte(s) of interest and the solubility of the analyte(s) (Rasmussen and Ahuja 2007; Skoog et al. 2004). Solvents such as methanol, isopropyl alcohol, chloroform, and some phosphate buffers at various pH values, as well as combinations of these solvents were tried. The mobile phase of methanol and dibasic phosphate buffer was tried in different proportions and different pH values. However, an isocratic mixture of methanol and 0.05 M dibasic phosphate buffer (pH 4.0) in the ratio of (30:70; v/v) was chosen as the mobile phase because it produced the best resolution of peaks, peak symmetry and separation of all components within the least retention times. Mean retention times of 2.4 ± 0.0, 4.2 ± 0.01 and 7.2 ± 0.0 were recorded for chlorpheniramine maleate, paracetamol and caffeine respectively as depicted in Fig. 1.

#### Stationary phase selection

The polarities of the analytes of interest were taken into consideration when choosing the stationary phase. As the drug molecules are polar or moderately polar, reversed phase stationary phases were tried (Sawyer and Kumar 2003; Chandra and Sharma 2013). A Phenomenex C18 reversed phase column (Luna 5µ, 250 × 4.6 mm) was chosen in order to reduce the time of interaction between the stationary phase and the analytes. This helped to reduce analysis time as there is reduced affinity of the analytes for the stationary phase, and increased interaction of the analytes with the mobile phase (Rasmussen and Ahuja 2007; Skoog et al. 2004).

### Chromatographic conditions

**Fig. 2:**
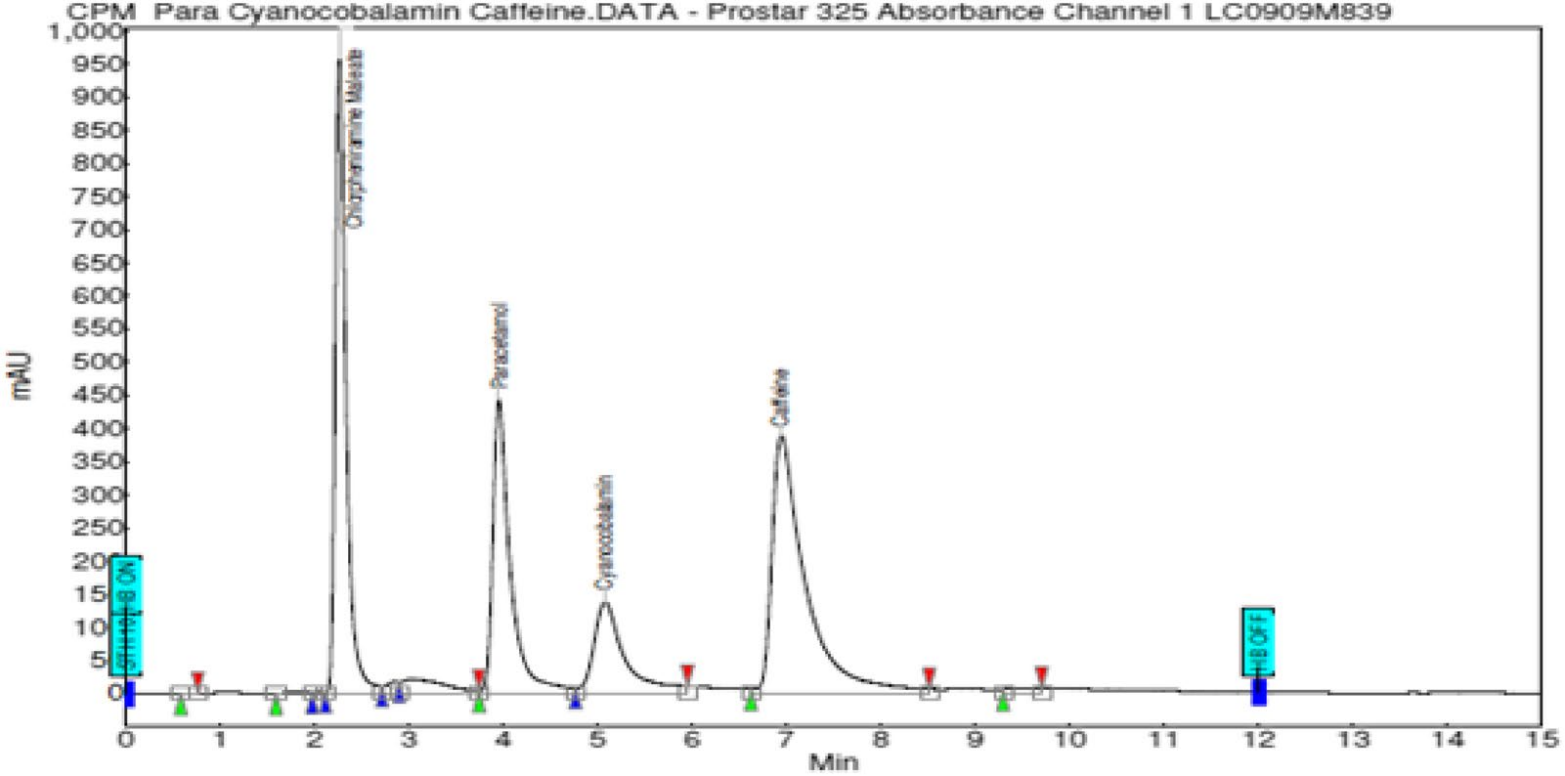
Chromatogram of single injection of solution containing standards of chlorpheniramine maleate, paracetamol, caffeine and internal standard (cyanocobalamine)

The established chromatographic conditions included a mobile phase of methanol: 0.05 M dibasic phosphate buffer (pH 4.0) (30:70; v/v), a C18 (Luna 5µ, 250 × 4.6 mm) stationary phase and a flow rate of 1 mL/min. Wavelength of detection was 215 nm and mode of elution was isocratic. Temperature was kept constant at 30 °C. These conditions gave the best resolution of peaks and separation of components. This is illustrated in Fig. 1. Figure 2 depicts the resolution of the internal standard from all the three APIs.

### Method validation

#### Linearity

Regression analysis of the standard calibration graphs was used to determine the linearity of the developed method. The results obtained showed that the method is linear for the three APIs in the range of 1–50, 1–500 and 1–150 μg/mL for CPM, PARA and CAF respectively with their coefficient of correlation (R^2^) all approximately equal to 1 (R^2^ = 1). The results are presented in Table 1.

**Table 1:** Calibration curve data for chlorpheniramine maleate, paracetamol and caffeine

Regression parameters	Chlorpheniramine maleate	Paracetamol	Caffeine
Regression equation	y = 0.7555x + 0.115	y = 0.5829x + 5.985	y = 1.7575x + 0.464
Correlation coefficient (R^2^)	0.9985	0.9997	0.9989
Slope	0.7555	0.5829	1.7575
Y-intercept	0.115	5.985	0.464
Concentration range (µg/mL)	1–50	1–500	1–150
Number of points	5	5	5

#### Accuracy

The results obtained from the determination of accuracy, expressed as percentage recovery, are summarized in Table 2. The mean recovery values of 100.76 ± 2.10 for chlorpheniramine maleate, 98.66 ± 1.72 for paracetamol and 100.13 ± 1.39 for caffeine depicts the accuracy of the method. The method was also accurate in the presence of tablet excipients as shown in Table 3. The mean recoveries and standard deviations at all three levels of determination were very good for all three APIs.

**Table 2:** Mean recovery of chlorpheniramine maleate, paracetamol and caffeine from the ternary mixture

Chlorpheniramine maleate			Paracetamol			Caffeine		
Amount injected (µg/mL)	Amount recovered (µg/mL)	% Recovery	Amount injected (µg/mL)	Amount recovered (µg/mL)	% Recovery	Amount injected (µg/mL)	Amount recovered (µg/mL)	% Recovery
6	6.17	102.80	120	118.09	98.40	12	12.09	100.50
8	7.89	98.60	160	156.61	97.09	16	15.77	98.60
10	10.09	100.90	200	200.89	100.50	20	5.07	101.30
		∑ = 100.76 SD = 2.10			∑ = 98.66 SD = 1.72			∑ = 100.13 SD = 1.39

**Table 3:** Mean recovery of chlorpheniramine maleate, paracetamol and caffeine in the presence of tablet excipients

Chlorpheniramine maleate		Paracetamol		Caffeine	
Amount added (µg/mL)	Mean recovery (%) ± SD	Amount added (µg/mL)	Mean recovery (%) ± SD	Amount added (µg/mL)	Mean recovery (%) ± SD
10	98.00 ± 1.30	50	102.2 ± 0.80	10	101.7 ± 1.10
20	100.95 ± 1.25	150	99.50 ± 1.50	20	100.75 ± 0.95
40	99.65 ± 0.95	300	100.90 ± 0.88	40	99.99 ± 1.25

These concentrations were injected in triplicates using 1.25 µg/mL of cyanocobalamine as internal standard

#### Precision

Precision of the developed method was determined based on inter and intra-day precisions. Results are presented in Table 4. The method was found to be precise since the RSD values for both inter-day and intra-day precision were below 2.0.

**Table 4:** Summary of validation parameters of chlorpheniramine maleate, paracetamol and caffeine

Parameter	Chlorpheniramine maleate	Paracetamol	Caffeine
Linearity (R^2^)	0.9985	0.9997	0.9989
Intra-day precision (% RSD)	0.42	0.31	0.60
Inter-day precision (% RSD)	0.18	0.11	0.37
LOD (µg/mL)	1.444 × 10^−2^	1.415 × 10^−4^	4.694 × 10^−5^
LOQ (µg/mL)	4.376 × 10^−2^	4.289 × 10^−4^	1.423 × 10^−4^
Accuracy (%)	100.76 ± 2.10	98.66 ± 1.72	100.13 ± 1.39
Robustness	Robust	Robust	Robust
Specificity	Specific	Specific	Specific

#### Selectivity

Each analyte chromatographic peak was not found to be attributable to more than one component which indicates that the method is selective. There were no interfering peaks from the tablet excipients. Selectivity of the current method was demonstrated by good resolution of the peaks of the three APIs and the internal standard as shown in Fig. 2.

### Limit of detection and limit of quantification

LOD and LOQ give a measure of the sensitivity of the developed method and the HPLC equipment (Varian Prostar Corporation, LC-20 AD single pump) used for the method development. The lowest amount of chlorpheniramine maleate, paracetamol and caffeine that could be detected were 1.444 × 10^−2^, 1.415 × 10^−4^, 4.694 × 10^−5^ µg/mL respectively. Also the lowest amount of chlorpheniramine maleate, paracetamol and caffeine that could be quantified were 4.376 × 10^−2^ µg/mL, 4.289 × 10^−4^, 1.423 × 10^−4^ µg/mL respectively. These values are very low and indicate that even very small amount of the analyte(s) can be detected using the developed method and the HPLC equipment under consideration. Hence the method and the equipment are very sensitive. The results are presented in Table 4.

### Robustness

Changes in mobile phase pH (±0.2 pH units), methanol composition in mobile phase (±2 %), wavelength (±2 nm), and temperature (±3°) did not adversely affect the developed method, meaning the developed method showed a high level of robustness. There were no significant difference between the results obtained with these changes in chromatographic conditions and those from the original chromatographic conditions.

### Specificity

Peak purities higher than 99 % were obtained for all three APIs in the chromatograms of sample solutions depicting that the method was very specific to the three APIs under consideration. There were no interfering peaks on the retention times of the APIs in the presence of excipients. This was very evident in the chromatograms of the tablet sample (Fig. 2).

### Selection of internal standard

Having developed and validated the method using external standards, the authors decided to go further to find an appropriate internal standard that could be used for the analysis of the three APIs. Even though external standard method is effective, internal standard method tends to yield the most accurate and precise results (Kupiec 2004). Cyanocobalamin was chosen as the best internal standard as its peak was very well resolved from that of all three APIs with its retention time falling in between those of paracetamol and caffeine. This is illustrated in Fig. 2. Calibration graphs of MPAR against concentration of each API were linear in the concentration ranges of 1–50, 1–500, and 1–150 μg/mL respectively for chlorpheniramine maleate, paracetamol and caffeine respectively. The regression coefficients, R^2^, were 0.9985, 0.9998 and 0.9988 respectively for CPM, PARA and CAF. Recovery values obtained (98–100 %) showed that the internal standard of cyanocobalamin is an excellent internal standard for determining the concentration of the APIs.

### Application

#### Analysis of marketed formulation

The developed method was used for the simultaneous quantification of the APIs in fixed dose combination tablets. To determine the content of CPM, PARA and CAF in commercial drugs, Coldrid™ tablets were analyzed with the proposed method and the results are presented in Table 5. The content (mg) and percentages of each API in the tablet sample was computed using peak areas and the regression equations from the calibration curves. The mean contents obtained for chlorpheniramine maleate, paracetamol and caffeine in the formulated product were in the acceptable range of 90–110 % of the label amount (BP 2009). The results show that the method is accurate in determining the content of the three active ingredients in fixed dose combination tablets. The mean contents of the three APIs were also computed using the internal standard, cyanocobalamine. The results are presented in Table 6.

**Table 5:** Assay data for marketed drug using external standard

Drugs	Label claim (mg)	Mean amount found (mg)	Mean content (%)
CPM	4.00	4.11	102.75 ± 0.80
PARA	500.00	500.21	100.04 ± 1.20
CAF	30.00	30.17	100.57 ± 1.15

**Table 6:** Assay data for marketed drug using internal standard of cyanocobalamine

Active pharmaceutical ingredient	Nominal concentration (µg/mL)	Mean peak area ratio	Mean actual concentration (µg/mL)	Mean content %
Chlorpheniramine maleate	0.100	0.098	0.098	98.0 ± 1.30
Paracetamol	12.500	1.022	12.775	102.2 ± 0.80
Caffeine	0.750	1.017	0.763	101.7 ± 1.10

## Conclusions

An accurate, simple, linear, specific and precise reversed phase HPLC method with UV detection for the simultaneous quantification of chlorpheniramine maleate, paracetamol and caffeine has been developed and validated. A second method employing internal standard was also proposed which is also very effective in determining the quantity of the three APIs in fixed dose combination drugs. The proposed method is rapid and convenient for laboratory quality control analysis of tablet dosage forms containing the three APIs. The brand of tablet containing the three APIs analyzed by the validated method had contents of the three APIs in the acceptable limits of the British Pharmacopoeia, and therefore showed adequate quality.

## Methods

### Materials and chemical

Reference standards of CPM (100.45 %) (Alta Labs Ltd, India), PARA (100.46 %) (Hebei Jiheng (group) Pharmaceutical Co. Ltd, China), and CAF (100.65 %) (AARTI Industries Ltd, India), cyanocobalamin (100.60) were obtained from the Food and Drugs Authority (FDA) in Ghana. Tablet formulation (COLDRID™) containing 4 mg CPM, 500 mg PARA and 30 mg CAF were obtained commercially. HPLC grade methanol (Fluka) was procured from the Chemical Store, KNUST. All chemical reagents were of analytical grade.

### Preparation of mobile phase

The mobile phase was a mixture of Phosphate buffer: methanol (70:30 v/v) (pH = 4.0). The mobile phase was prepared by mixing 700 mL of the phosphate buffer with 300 mL of methanol to produce 1 dm^3^ of mobile phase.

### Preparation of buffer solution

A phosphate buffer solution of pH 4.0 and of the buffer strength of 50 mM (0.05 M) was prepared by weighing accurately 0.68 g of potassium dihydrogen phosphate (KH_2_PO_4_) and dissolving it in 100 mL redistilled water and adjusting to pH 4.0 with 85 % orthophosphoric Acid (H_3_PO_4_).

### Preparation of standard solutions

Standard stock solutions of CPM, PARA and CAF were prepared by accurately weighing and dissolving 10 mg of chlorpheniramine maleate, 100 mg of paracetamol and 20 mg of Caffeine, in redistilled water to form a 100 mL of solution containing 100 µg/mL of chlorpheniramine maleate, 1000 µg/mL of paracetamol and 200 µg/mL of caffeine. Standard solutions of concentrations ranging from 2 to 10 µg/mL for chlorpheniramine maleate, 40–200 µg/mL for paracetamol and 4–20 µg/mL for caffeine were prepared from the stock solution for standard curves.

The internal standard solution was prepared by weighing 1 mg of the internal standard (cyanocobalamine) and dissolving in distilled water to form 100 mL of solution, with concentration of 10 µg/mL of cyanocobalamin.

### Preparation of sample solution

Twenty (20) tablets of coldrid™ tablets were powdered and 150 mg equivalent of the powdered sample was taken and dissolved in mobile phase to produce 100 mL of solution of concentration 1500 µg/mL.

1.0 mL of the stock solution was pipette into a 100 mL volumetric flask and 12.5 mL of the stock solution of internal standard (cyanocobalamine) added and made up to the mark with distilled water. The final concentration of chlorpheniramine maleate, paracetamol, caffeine and cyanocobalamine were 0.10, 12.50, 0.75 and 1.25 µg/mL respectively in the resultant solution. The resultant solution was then injected into the LC system. Three replicate injections were performed and the average percentage content calculated.

### Instrumentation and chromatographic condition

A high performance liquid chromatographic system Varian Prostar 325 (Varian Prostar Corporation, LC-20 AD single pump) with autosampler and Varian Prostar SPD-20A UV/VIS detector was used for analysis. Separation was carried out at 30 °C, using an octadecylsilane (ODS), Phenomenex Luna, C18 (2) 100A, 250 × 4.6 mm, 5µ analytical column.

### Internal standard selection

Various compounds that do not react with the three APIs were mixed with standard solutions of the three APIs and ran using the established chromatographic conditions. The compound that produced a peak that was well resolved from those of the APIs was chosen as the internal standard.

### Obtaining concentration of APIs using internal standard

Various concentrations of the APIs were injected together with known concentrations of the internal standard and the mean peak area ratios (MPAR) were recorded. Calibration graphs of MPAR against concentration of each API was plotted to relate peak area ratios to concentrations (Kupiec 2004; Okine et al. 2006). The actual concentrations of the APIs were interpolated from the required calibration graphs using the respective MPAR. Similarly, recovery was performed by injecting mixtures containing known concentrations of the three APIs and the internal standard and the MPAR was used to verify the concentrations of the APIs.

### Method validation

The method was validated based on International Conference on Harmonization (ICH) guidelines (ICH 2005). Validation parameters included accuracy, linearity, precision, specificity, robustness, limit of detection (LOD) and limit of quantification (LOQ).

### Linearity

Standard calibration graphs for the APIs were obtained by plotting peak areas produced by injection of standard solutions against the concentrations used. The calibration graphs were analyzed by regression analysis. The equation, correlation coefficient, slope, and y-intercept were then calculated.

### Accuracy

The accuracy of method was determined by studying recovery at three different concentrations for all active pharmaceutical ingredients, by replicate analysis (n = 3). Samples of known concentration (reference standard solutions) were analyzed and the measured values, from the respective peak areas, were compared with the true values.

### Precision

Precision of the developed method was determined based on inter and intra-day precisions. The intra-day precision was evaluated by analysing six sample solutions (n = 6) at three concentration levels of 10, 20 and 50 µg/mL for CPM, 10, 50 and 100 µg/mL for PARA and 10, 20 and 50 µg/mL for CAF, and calculating the actual concentrations of these standard solutions. Three replicate injections were performed for each sample within a day and the mean concentrations determined. The inter-day precision was evaluated in three consecutive days (n = 18) using the same concentrations of the APIs as in the intra-day precision. The concentrations of the three APIs were determined and relative standard deviations (RSD) were calculated.

### Selectivity

Selectivity of the method was assessed by the peak purity test. Each analyte chromatographic peak must not be found to be attributable to more than one component.

### Limit of detection and limit of quantification

The LOD and LOQ of the developed method were determined by progressively injecting low concentrations of the standard solutions using the method developed. The smallest amount of the analyte which produces a measurable response is the LOD (signal-to-noise ratio of 3). The LOQ is the lowest concentration of the analyte which gives a response that be quantified accurately (signal-to-noise ratio of 10).

### Robustness

Robustness of the method was assessed by examining changes in different experimental conditions. Six sample solutions were prepared and analyzed using the established conditions and by varying some of the chromatographic conditions. Changes in mobile phase pH (±0.2 pH units), methanol composition in mobile phase (±2 %), wavelength (±2 nm), and temperature (±3°) were made and data obtained. Data obtained was subjected to statistical analysis using analysis of variance (ANOVA test).

### Specificity

Specificity of the developed method was evaluated by preparing a solution of the reference standards of the three APIs in the presence of excipients. Five injections of this solution were carried out to observe any interfering peaks.
